# Acclimation of Biochemical and Diffusive Components of Photosynthesis in Rice, Wheat, and Maize to Heat and Water Deficit: Implications for Modeling Photosynthesis

**DOI:** 10.3389/fpls.2016.01719

**Published:** 2016-11-22

**Authors:** Juan A. Perdomo, Elizabete Carmo-Silva, Carmen Hermida-Carrera, Jaume Flexas, Jeroni Galmés

**Affiliations:** ^1^Plant Biology and Crop Science, Rothamsted ResearchHarpenden, UK; ^2^Research Group on Plant Biology under Mediterranean Conditions, Universitat de les Illes BalearsPalma, Spain; ^3^Lancaster Environment Centre, Lancaster UniversityLancaster, UK

**Keywords:** photosynthesis, high temperature, water deficit, crops, C_3_, C_4_

## Abstract

The impact of the combined effects of heat stress, increased vapor pressure deficit (VPD) and water deficit on the physiology of major crops needs to be better understood to help identifying the expected negative consequences of climate change and heat waves on global agricultural productivity. To address this issue, rice, wheat, and maize plants were grown under control temperature (CT, 25°C, VPD 1.8 kPa), and a high temperature (HT, 38°C, VPD 3.5 kPa), both under well-watered (WW) and water deficit (WD) conditions. Gas-exchange measurements showed that, in general, WD conditions affected the leaf conductance to CO_2_, while growth at HT had a more marked effect on the biochemistry of photosynthesis. When combined, HT and WD had an additive effect in limiting photosynthesis. The negative impacts of the imposed treatments on the processes governing leaf gas-exchange were species-dependent. Wheat presented a higher sensitivity while rice and maize showed a higher acclimation potential to increased temperature. Rubisco and PEPC kinetic constants determined *in vitro* at 25°C and 38°C were used to estimate V_cmax_, J_max_, and V_pmax_ in the modeling of C_3_ and C_4_ photosynthesis. The results here obtained reiterate the need to use species-specific and temperature-specific values for Rubisco and PEPC kinetic constants for a precise parameterization of the photosynthetic response to changing environmental conditions in different crop species.

## Introduction

Mean global air temperatures are predicted to rise on average 0.3–0.6°C per decade over the next century, with heat waves becoming more frequent, intense and persistent (IPCC, 2013). In certain geographical regions, increased annual temperatures and heat wave frequency might be accompanied by decreased precipitation, causing decreased water availability for plants. While predicted increases in the concentration of atmospheric CO_2_ may be positive for plant productivity (Long et al., 2006), in some agricultural regions these beneficial effects are likely to be offset by negative impacts of increased temperature and water deficit (Gornall et al., 2010). Hence, future predicted environments will compromise agricultural productivity and food security for the increasing world population. A more detailed understanding of the capacity of major crops, which sustain most of the human caloric intake, to respond and acclimate to water deficit and high temperature is key to mitigate the negative impacts of climate change on plant productivity.

Decreased crop productivity under water deficit and high temperature tend to be primarily caused by limited photosynthetic carbon assimilation and persisting mitochondrial respiration (Atkin et al., 2006; Flexas et al., 2006a; Ainsworth and Ort, 2010). Under water deficit, stomatal conductance (g_s_) decreases, minimizing water loss, with parallel decreases in mesophyll conductance (g_m_) (Flexas et al., 2013; Théroux-Rancourt et al., 2014). As a consequence, the capacity of the leaf to transfer CO_2_ from the atmosphere to the sites of carboxylation in the chloroplast stroma decreases under drought conditions (Galmés et al., 2011). On the other hand, for most species, the photosynthetic machinery is robust enough to be largely unaffected by conditions of mild to moderate water deficit (Flexas et al., 2006a; Galmés et al., 2007b, 2013). Therefore, limitations in water availability decrease CO_2_ assimilation mainly through diffusive rather than metabolic limitations (Flexas et al., 2006b; Galmés et al., 2007b). Increased temperature often results in increased vapor pressure deficit (VPD), which may exacerbate even more the diffusional limitations (Perez-Martin et al., 2009).

Photosynthetic processes are strongly temperature dependent, and moderate increases above the thermal optimum cause decreases in photosynthetic CO_2_ uptake. Contrarily to water deficit, the negative impact of high temperature on the rate of net CO_2_ assimilation (A_N_) is mostly due to biochemical limitations (Scafaro et al., 2011; Carmo-Silva et al., 2012). Rubisco activase is extremely heat-sensitive and this results in deactivation of Rubisco catalytic sites at moderately high temperatures (Salvucci and Crafts-Brandner, 2004; Yamori et al., 2011). Moreover, increases in the maximum catalytic rate of carboxylation (k_cat_^c^) with temperature are offset by decreases in the affinity of Rubisco for CO_2_ (i.e., increases in the Michaelis–Menten constant for CO_2_, K_c_, and decreases in the specificity factor, S_c/o_) and the lower CO_2_/O_2_ ratio in solution, which increase photorespiration (Sage and Kubien, 2007). On the other hand, mitochondrial respiration appears to be relatively unaffected by water availability but increases with temperature compared to photosynthesis (Atkin and Tjoelker, 2003; Galmés et al., 2007c; Atkin and MacHerel, 2009; Rodríguez-Calcerrada et al., 2010; Silim et al., 2010). Thus, the response of mitochondrial respiration to combined heat and drought would tend to further decrease the leaf carbon balance.

The above responses correspond to general trends observed when stresses are applied over relatively short periods of 15–20 days. In nature plants face long-term exposure to water deficit and high temperature, and photosynthesis and mitochondrial respiration have been shown to acclimate to both water (Walters, 2005; Galmés et al., 2006; Flexas et al., 2009) and heat stress (Berry and Björkman, 1980; Yamori et al., 2005; Campbell et al., 2007; Sage and Kubien, 2007), although the capacity and mechanisms of plant acclimation may differ between species (Hikosaka et al., 2006; Kattge and Knorr, 2007; Dillaway and Kruger, 2011; Scafaro et al., 2011; Cheesman and Winter, 2013). In semi-arid climates like the Mediterranean, drought, and heat stress occur simultaneously and exert a combined effect on plant functioning (Mittler, 2006).

Most mechanistic models of carbon uptake and release in C_3_ and C_4_ leaves currently do not account for long-term responses to changes in the environmental conditions (e.g., von Caemmerer, 2000; Pittermann and Sage, 2001; Hu et al., 2010). Further, these models are usually parameterized with invariable values and temperature responses for the Rubisco kinetic parameters and g_m_ among different species. In fact, the use of Rubisco kinetics and g_m_ values experimentally measured in *Nicotiana tabacum* have been employed in most of the studies modeling leaf gas exchange responses to variations in the environment (Bernacchi et al., 2001, 2002, 2003; Diaz-Espejo, 2013; von Caemmerer, 2013). However, Rubisco kinetic constants and g_m_, as well as their dependence on temperature vary among species. These species-specific differences in Rubisco parameters explain differences in photosynthetic responses to temperature, and significantly bias modeling of C_3_ photosynthesis (Diaz-Espejo, 2013; Walker et al., 2013; Galmés et al., 2014; Flexas and Díaz-Espejo, 2015; von Caemmerer and Evans, 2015). Further, C_4_ photosynthesis modeling at variable temperatures has received little attention (Massad et al., 2007; Sage and Kubien, 2007; von Caemmerer, 2013). It is important that future approaches incorporate the temperature dependence of phosphoenolpyruvate carboxylase (PEPC) activity and the thermal response of the underlying kinetic parameters, such as the affinity of PEPC for CO_2_ (K_P_).

Rice (*Oryza sativa*), wheat (*Triticum aestivum*), and maize (*Zea mays*) are major commercially important crops. Together, these species account for ~85% of global cereal production and contribute the majority of the energy in food of humans eaten directly as staple foods or indirectly through consumption of livestock fed with grain (Grassini et al., 2013). The three cereals were domesticated in different climates and differ largely in their growth environments: rice and maize are cultivated in tropical hot and wet climates, whereas wheat tends to be grown in cooler temperate climates (Makino, 2011). Further, these species differ in their photosynthetic mechanism, maize is a C_4_ crop, and rice and wheat are C_3_ crops. The objectives of the present study were: (i) to analyze the patterns of response of leaf photosynthesis and respiration to long-term drought, VPD, and temperature stress in these three crops; (ii) to compare the sensitivity and acclimation capacity of leaf photosynthesis and respiration to these stresses in the three species; and (iii) to compare the effect on C_3_ and C_4_ photosynthetic models of using species-specific kinetics of Rubisco and PEPC, and their species-specific response to temperature.

## Materials and methods

### Plant material, growth conditions, and treatments

Plants of rice (*O. sativa* L. cv. Bomba), wheat (*T. aestivum* L. cv. Cajeme), and maize (*Z. mays* cv. Carella) were grown from seeds in a greenhouse in 3.5 L pots containing a 70:30 mixture (v:v) of horticultural substrate (Projar S.A, Spain) and perlite (granulometry A13, Projar S.A, Spain). After 2 weeks, seedlings were selected to uniform size and were moved to a controlled environment room. Light was provided by metal halide lamps (OSRAM, Germany) placed at specific distances from the plants to obtain a photosynthetically active photon flux density (PPFD) of 500 μmol m^−2^ s^−1^, with a photoperiod of 12 h day/12 h night. Ambient temperature and relative humidity were monitored with portable sensors Testo 175-H1 data logger (Gerilab, Spain). Relative humidity (RH) was maintained at 40–60% using humidifiers. For logistical reasons, assays were performed in two consecutive experiments with two sets of plants of identical age. For the first experiment a first set of plants of the three species was grown under control conditions (CT, 25/20°C day/night), which combined with the set RH resulted in a VPD of 1.8/1.0 kPa day/night. A second set of plants, for the second experiment, was grown at higher temperature (HT, 38/33°C, resulting in VPD 3.5/2.3 kPa day/night). Only temperature and VPD differed between the two sets of plants or experiments, while all other environmental conditions (e.g., light intensity and quality, air removal, photoperiod duration) were identical and computer-controlled.

For each set of plants, i.e., for each growing temperature and VPD treatment, 10 pots per species were grown at soil field capacity until plants presented fully expanded leaves (typically 2 weeks). Thereafter, 20 days after germination, pots of all species were randomly assigned to two irrigation treatments: five pots per species were maintained at field capacity throughout the experiment (well-watered treatment, WW) and five were maintained at 45% of field capacity (moderate water deficit treatment, WD). The level of water availability was determined gravimetrically by weighing the pots daily and maintained by compensating water losses with 50% Hoagland's solution. Plants were considered to be under water deficit when g_s_ was decreased by 40% compared to the well-watered plants; g_s_ was considered as a good indicator of the water deficit status, as previously demonstrated (Medrano et al., 2002).

New leaves were allowed to develop and expand under the two irrigation treatments for a minimum of 30 days. All measurements and samples were taken 40–50 days after the water treatment was initiated (i.e., 60–70 days after germination), on new leaves developed completely under the temperature and/or water treatments (Perdomo, 2015).

### Gas exchange and chlorophyll *a* fluorescence measurements

Leaf gas exchange and chlorophyll *a* fluorescence measurements were performed with a portable photosynthesis system (Li-6400; Li-Cor Inc., USA) equipped with a leaf chamber fluorometer (Li-6400-40, Li-Cor Inc., USA), the latter using the multi-flash protocol (Loriaux et al., 2013). The response of net CO_2_ assimilation rate (A_N_) to varying intercellular airspace CO_2_ concentration (C_i_) was measured on the youngest fully expanded leaf at a saturating photosynthetic active radiation (PAR) of 1500 μmol m^−2^ s^−1^ (10% blue light), a relative humidity of the incoming air between 40 and 50% and at two leaf temperatures: 25°C and 38°C. A_N_-C_i_ curves were initiated by allowing the leaf to reach steady-state A_N_ and stomatal conductance (g_s_) at a CO_2_ concentration in the leaf chamber (C_a_) of 400 μmol CO_2_ mol^−1^ air, before varying the C_a_ between 50 and 2000 μmol CO_2_ mol^−1^ air. Corrections for the leakage of CO_2_ into and out of the leaf chamber were applied to all gas-exchange data (Flexas et al., 2007).

The photochemical efficiency of photosystem II (Φ_PSII_) was determined according to Genty et al. (1989):

(1) $$\Phi_{\text{PSII}}=(\text{F}\prime_{\text{m}}{\text{-F}}_{\text{s}})/\text{F}\prime_{\text{m}}$$

where F_s_ is the steady-state fluorescence yield and ${\text{F}}_{\text{m}}^{\prime }$ the maximum fluorescence yield obtained with a light-saturating pulse of 8000 μmol m^−2^ s^−1^.

Φ_PSII_ was used for the calculation of the linear rate of electron transport (ETR) according to Krall and Edwards (1992):

(2) $$\text{ETR}=\Phi_{\text{PSII}}\cdot \text{PPFD}\cdot \alpha \cdot \beta$$

where α is the leaf absorptance and β is the partitioning of absorbed quanta between photosystems I and II. β was assumed to be 0.5 (Laisk and Loreto, 1996; Tosens et al., 2012). α was measured for all species grown under each treatment inside a dark chamber using the light source from the Li-6400 and a spectroradiometer (HR2000CG-UV-NIR; Ocean Optics Inc., USA), as described by Schultz (1996). All values obtained for α were 0.86–0.87, with non-significant differences between species and species × treatment combinations.

### Modeling C_3_ photosynthesis in wheat and rice

From combined gas-exchange and chlorophyll *a* fluorescence measurements, mesophyll conductance to CO_2_ (g_m_) was estimated for wheat and rice according to the variable J method (Harley et al., 1992):

(3) $$\begin{array}{l} {\text{g}}_{\text{m}}={\text{A}}_{\text{N}}/({\text{C}}_{\text{i}}-(\Gamma^{*}(\text{ETR}+8(A_{\text{N}}+{\text{R}}_{\text{L}}))\\ \;/(\text{ETR}-4({\text{A}}_{\text{N}}+{\text{R}}_{\text{L}})))) \end{array}$$

where A_N_ and C_i_ were obtained from gas exchange measurements at saturating light. The rate of non-photorespiratory CO_2_ evolution in the light (R_L_) was determined as half of the mitochondrial respiration at pre-dawn (R_dark_), which was measured at a C_a_ of 400 μmol CO_2_ mol^−1^ air and leaf temperatures of 25°C or 38°C. The chloroplast CO_2_ compensation point in the absence of mitochondrial respiration (Γ^*^) was calculated from the *in vitro* measurements of Rubisco specificity factor (S_c/o_) as:

(4) $$\Gamma^{*}\;=\;\frac{0.5\;O}{S_{\text{c/o}}}$$

A_N_-C_i_ curves were converted into A_N_-C_c_ curves using the values of g_m_:

(5) $${\text{C}}_{\text{c}}={\text{C}}_{\text{i}}-({\text{A}}_{\text{N}}/{\text{g}}_{\text{m}})$$

Maximum velocity of Rubisco carboxylation (V_cmax_) and maximum electron transport rate (J_max_) were calculated from A_N_-C_c_ curves according to Bernacchi et al. (2002), but using the Rubisco kinetic constants (the Michaelis–Menten constants for CO_2_ and O_2_ and the S_c/o_) measured for each species at 25°C and 38°C. For comparative purposes, V_cmax_ and J_max_ were also calculated for rice and wheat using the values for the Rubisco kinetics parameters and respective temperature dependencies reported for tobacco by Bernacchi et al. (2001, 2002).

### Modeling C_4_ photosynthesis in maize

The C_4_ photosynthesis model described by von Caemmerer (2000) was applied to the A_N_-C_i_ curves measured for maize as detailed by Massad et al. (2007), with the modifications of Carmo-Silva et al. (2008). The maximum velocity of Rubisco carboxylation (V_cmax_) and the maximum velocity of PEPC carboxylation (V_pmax_), as well as the CO_2_ concentrations in the bundle sheath (C_s_) and in the mesophyll cells (C_m_) were estimated from the hyperbolic function describing the A_N_-C_i_ curves using a C_i_ step-size of 5 μmol mol^−1^, by applying the equations:

(6) $${\text{A}}_{\text{N}}=g_{\text{m}}\left(C_{i}-C_{\text{m}}\right)$$

(7) AN=CsVcmaxCs+Kc(1+OKo)(1−Γ*OCs)−RL

(8) $${\text{A}}_{\text{N}}=\frac{C_{\text{m}}V_{\text{pmax}}}{C_{\text{m}}\;+\;K_{\text{p}}}\;-\;g_{\text{bs}}\left(C_{\text{s}}-C_{\text{m}}\right)-R_{\text{m}}$$

In these equations, the oxygen partial pressure in the bundle sheath and mesophyll cells (O), the bundle sheath conductance to CO_2_ (g_bs_), and the mesophyll conductance to CO_2_ (g_m_) were assumed to be invariable between water and temperature treatments, as in Carmo-Silva et al. (2008) and Massad et al. (2007), respectively. Constant values for these parameters were O = 210 mbar, g_bs_ = 3 mmol m^−2^ s^−1^, and g_m_ = 2 mol m^−2^ s^−1^ (von Caemmerer, 2000).

The model also requires values for kinetic constants of Rubisco and PEPC: the Rubisco specificity for CO_2_/O_2_ (S_c/o_, from which Γ^*^ is calculated as 0.5 O/S_c/o_), the Michaelis–Menten constants of Rubisco for CO_2_ (K_c_) and O_2_ (K_o_), and the Michaelis–Menten constant of PEPC for CO_2_ (K_p_). V_cmax_ and V_pmax_ values calculated using the *in vitro* kinetic constants of maize Rubisco and PEPC at 25°C and 38°C were compared to V_cmax_ and V_pmax_ calculated using the values at 25°C for Γ^*^ (0.000193), K_c_ (65 Pa) K_o_ (45 kPa), and K_p_ (8 Pa) reported in von Caemmerer (2000). The temperature equations provided by Bernacchi et al. (2001, 2002) were used to calculate values for the Rubisco kinetic constants at 38°C, while K_p_ was assumed to be invariable with temperature changes.

### Determination of michaelis–menten constants of rubisco and PEPC for their gaseous substrates

The Michaelis–Menten constants of Rubisco for CO_2_ (K_c_) and O_2_ (K_o_) were determined at 25°C and 38°C using leaf samples of rice, wheat, and maize, as previously described Galmés et al. (2015). In the present study, assays were done under either 0% O_2_ (100% N_2_) or 21% O_2_ (in 79% N_2_), and thus K_o_ was estimated using the equation:

(9) $${\text{K}}_{\text{c}}(21\%{\text{O}}_{2})={\text{K}}_{\text{c}}(0\%{\text{O}}_{2})\cdot (1+[{\text{O}}_{2}]/{\text{K}}_{\text{o}})[9]$$

TheMichaelis–Menten constant of PEPC for CO_2_ (K_p_) was determined for maize at 25°C and 38°C, essentially as described by Uedan and Sugiyama (1976). PEPC was extracted from leaf samples (1.2 cm^2^) by grinding in a mortar with 46 mg insoluble PVPP and 2 mL of ice-cold extraction buffer containing 50 mM Bicine-NaOH (pH 8.2), 1 mM EDTA, 0.18% (w/v) PEG4000, 11 mM ε-aminocaproic acid, 2.2 mM benzamidine, 1.8 mg bovine serum albumin (BSA), 2.8% (v/v) Tween, and 1.8 mM Na_2_H_2_PO_4_. The homogenate was centrifuged for 4 min at 13,000 *g* and 4°C. Eight 7 mL septum-sealed vials containing 990 μL assay buffer [50 mM Bicine-NaOH (pH 8.2), 5 mM MgCl_2_, 1 mM EDTA, 1 mM DTT, 100 mM phosphoenolpyruvate (PEP), 20 mM NADH, 100 mM malic dehydrogenase (MDH), 100 mM glucose-6-phospate] and varying concentrations of NaH^14^CO_3_ (0–10 mM, 1.3 × 10^10^ Bq mol^−1^) were equilibrated with nitrogen (N_2_) for 30 min. Reactions were started by the addition of 10 μl leaf extract and quenched after 1 min with 10 M formic acid. Acid stable ^14^C was measured by liquid scintillation counting. To convert K_c_, K_o_, and K_p_ values from concentration to partial pressures, solubilities for CO_2_ of 0.0334 mol (L bar)^−1^ at 25°C and 0.0243 mol (L bar)^−1^ at 38°C and for O_2_ of 0.00126 mol (L bar)^−1^ at 25°C and 0.00102 mol (L bar)^−1^ at 38°C were used.

### Rubisco specificity factor determination

Rubisco specificity for CO_2_/O_2_ (S_c/o_) was measured at 25°C and 38°C for rice, wheat, and maize (*n* = 6–12) using purified leaf extracts obtained as in Galmés et al. (2006) and the oxygen electrode (Model DW1; Hansatech, Kings Lynn., UK) method described by Parry et al. (1989). Reaction mixtures contained (final concentrations) 100 mM Bicine-NaOH (pH 8.2), 10 mM MgCl_2_, 0.15 mg mL^−1^ carbonic anhydrase, 2 mM NaH^14^CO_3_ (18.5 kBq mol^−1^), activated Rubisco from purified extracts (20 μL), and 2.5 μM RuBP. The basic buffer was pre-equilibrated with CO_2_-free air at the temperature of measurement. RuBP oxygenation was calculated from the oxygen consumption and carboxylation from the amount of ^14^C incorporated into PGA when all the RuBP had been consumed. To convert the S_c/o_ values from concentration to partial pressures, the CO_2_ and O_2_ solubilities were used as described above for the Rubisco and PEPC kinetics.

### Temperature/VPD sensitivity and acclimation

The effect of temperature/VPD on the main leaf gas exchange parameters was examined using two indexes. The temperature sensitivity index (TSI), to assess the impact of an increase in the measuring temperature on a given parameter (Y) in plants grown at 25°C (CT), was calculated as:

(10) $$TSI=\;\frac{Y_{\text{CT}-25}\;}{Y_{\text{CT}-38}\;}$$

The temperature acclimation index ratio (TAI) of the same leaf gas exchange parameters measured and grown at a specific temperature (Silim et al., 2010) was calculated as:

(11) $$TAI=\;\frac{Y_{HT-38}\;}{Y_{CT-25\;}\;}$$

### Statistical analysis

The statistical significance of trait variation was tested by factorial ANOVA, with species, irrigation and temperature/VPD regimes, and the interaction between treatments, as fixed factors. *Post hoc* comparison between treatments was performed with Duncan test (*P* < 0.05) using Statistica 6.0 software package (StatStof Inc., USA). Regressions coefficients were calculated with Sigma Plot 11.0 software package.

## Results

### Leaf CO_2_ conductances and assimilation in rice, wheat, and maize grown under water deficit and elevated temperature and VPD

**Figure 1:**
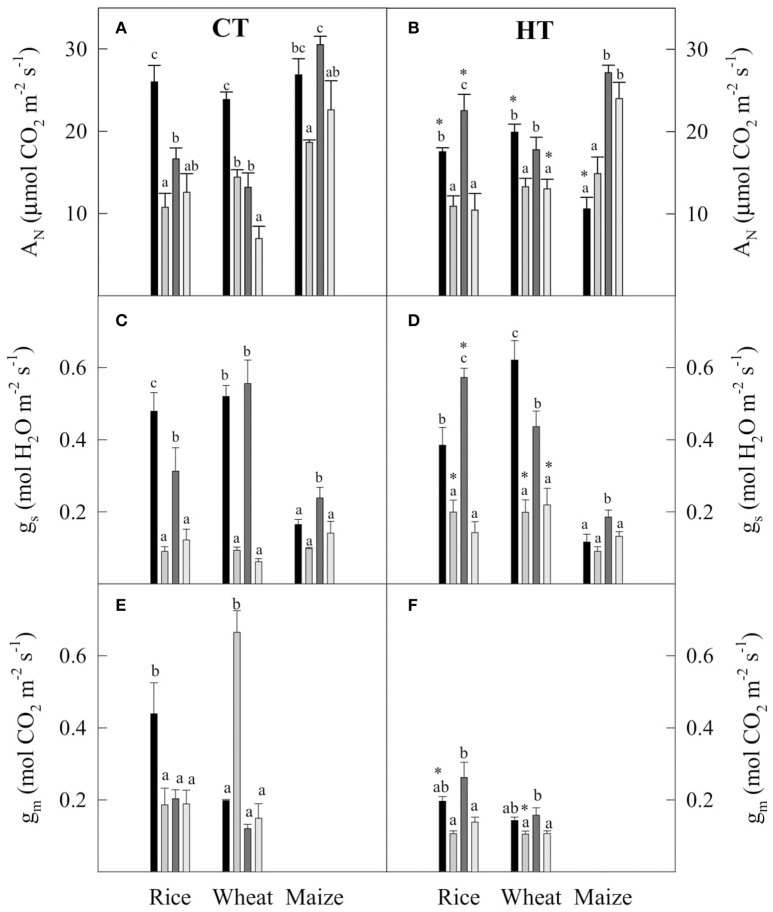
**(A,B)** The net CO_2_ assimilation rate (A_N_), **(C,D)** the stomatal conductance (g_s_) and **(E,F)** the mesophyll conductance (g_m_) in plants grown at CT **(A,C,E)** and HT **(B,D,F)** for rice, wheat, and maize measured at 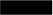 WW-25°C, 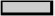 WD-25°C, 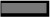 WW-38°C, and 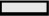 WD-38°C. Values are means ± standard error (*n* = 3–5). Different letters denote statistically significant differences between treatments within each species and growth temperature and asterisks between the two growth temperatures within the same species, irrigation treatment, and temperature of measurement (Duncan analysis, *P* < 0.05).

Plants of rice, wheat, and maize grown at 25°C and 1.8 kPa VPD with optimal water supply (CT-WW) had similar values of net CO_2_ assimilation rate (A_N_) at 25°C (Figure 1A). By comparison, when A_N_ was measured at 38°C in the same plants it had similar values to that in maize at 25°C in maize, but was decreased slightly in rice and substantially in wheat. In plants grown at 38°C and 3.5 kPa VPD with optimal water supply (HT-WW), A_N_ measured at 38°C was higher in maize than in rice or wheat, and A_N_ measured at 25°C was largely decreased in maize, and slightly decreased in rice and wheat as compared to measurements at the higher temperature (Figure 1B).

Growth under conditions of water deficit (CT-WD and HT-WD) had a negative impact on A_N_ for all plants except for maize grown at HT, mostly as a consequence of decreased stomatal conductance (g_s_, Figures 1C,D). Effects of water deficit and growth temperature on mesophyll conductance (g_m_) estimated for the C_3_ species showed less obvious trends (Figures 1E,F). Comparison of the results for g_m_, obtained with the three different methods (Figure S1), showed some scattering in the data, but significant positive correlations (*P* < 0.01) were obtained between the method of Harley (Harley et al., 1992; adopted in this work for subsequent comparisons and modeling) and two alternative methods (Ethier and Livingston, 2004; Yin et al., 2009). No clear pattern was observed for the 4 treatments out of 16 showing discrepancies, e.g., in some cases measurements were at 25°C and in others at 38°C. The unexpected increase in g_m_ in wheat plants grown at 25°C under WD compared to WW conditions was confirmed by the three estimation methods (Figure S1).

Decreases in g_s_ and g_m_ largely explained the limitation of A_N_ in rice and wheat plants under WD conditions (Figure 1), so that a tight correlation was observed between the total leaf conductance to CO_2_ (g_t_, calculated from integration of g_s_ and g_m_) and A_N_ (Figure S2). A similar trend was observed for g_s_ in maize (Figure S2), supporting the conclusion that diffusive limitations to photosynthesis predominate in plants exposed to moderate water deficit conditions. Conversely, in wheat plants grown at 25°C and measured at 38°C, A_N_ was much decreased even though g_t_ was mostly unaffected (Figure S2).

### Leaf mitochondrial dark respiration (R_dark_) in rice, wheat, and maize grown under water deficit and elevated temperature and VPD

**Figure 2:**
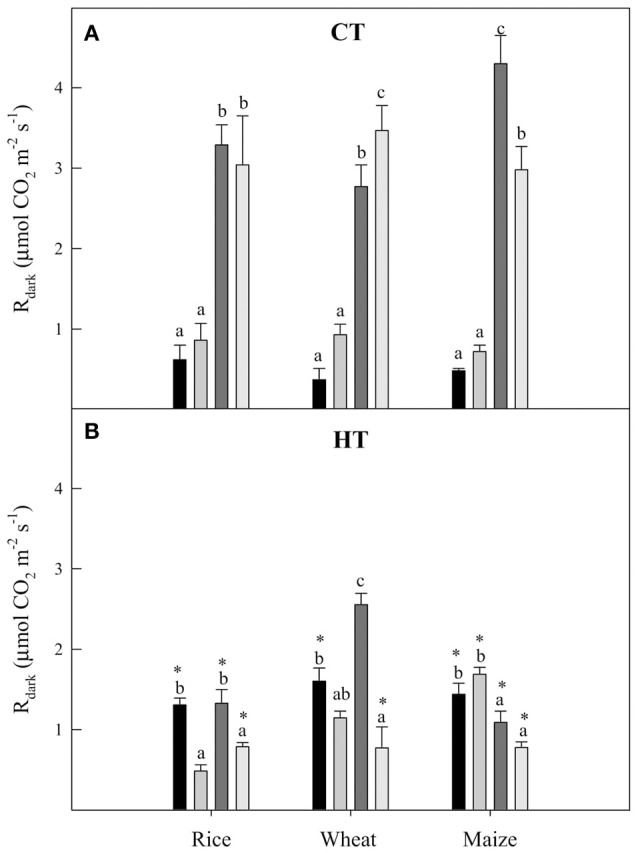
**The mitochondrial respiration at pre-dawn (R_dark_) in plants grown at CT (A)** and HT **(B)** for rice, wheat, and maize measured at 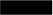 WW-25°C, 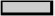 WD-25°C, 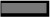 WW-38°C, and 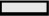 WD-38°C. Values are means ± standard error (*n* = 3–5). Different letters denote statistically significant differences among treatments within each species and same growth temperature and asterisks between the two growth temperatures within the same species, irrigation treatment and temperature of measurement (Duncan analysis *P* < 0.05).

Plants of all three species grown at CT showed similar responses of mitochondrial dark respiration rate (R_dark_) to the imposed treatments (Figure 2). These responses consisted of a boost of R_dark_ after a sudden increase in the temperature of measurement. The effects of WD on R_dark_ in CT plants were non-significant at the measurement temperature of 25°C in all three species, but became significant in wheat and maize measured at 38°C. In HT grown plants, the patterns of response of R_dark_ to the imposed treatments were radically different to those displayed by CT plants. In HT plants, R_dark_ became sensitive to the irrigation treatment in the C_3_ crops (except for wheat measured at 25°C), but not in maize. In addition, in HT plants the effects of the measuring temperature on R_dark_ were less evident than in CT plants, with significant changes only observed in maize and in HT-WW wheat.

### Long-term effects of water deficit and high temperature and VPD stress on the photosynthetic biochemistry of the three crops

The response of photosynthesis to increasing CO_2_ concentration was analyzed in the three species on the basis of the CO_2_ concentration in the chloroplastic stroma (i.e., A_N_–C_c_ curves in rice and wheat, and A_N_–C_s_ in maize). All crops displayed the well-described response of A_N_ to increasing C_c_ or C_s_ (Figures S3–S5).

**Figure 3:**
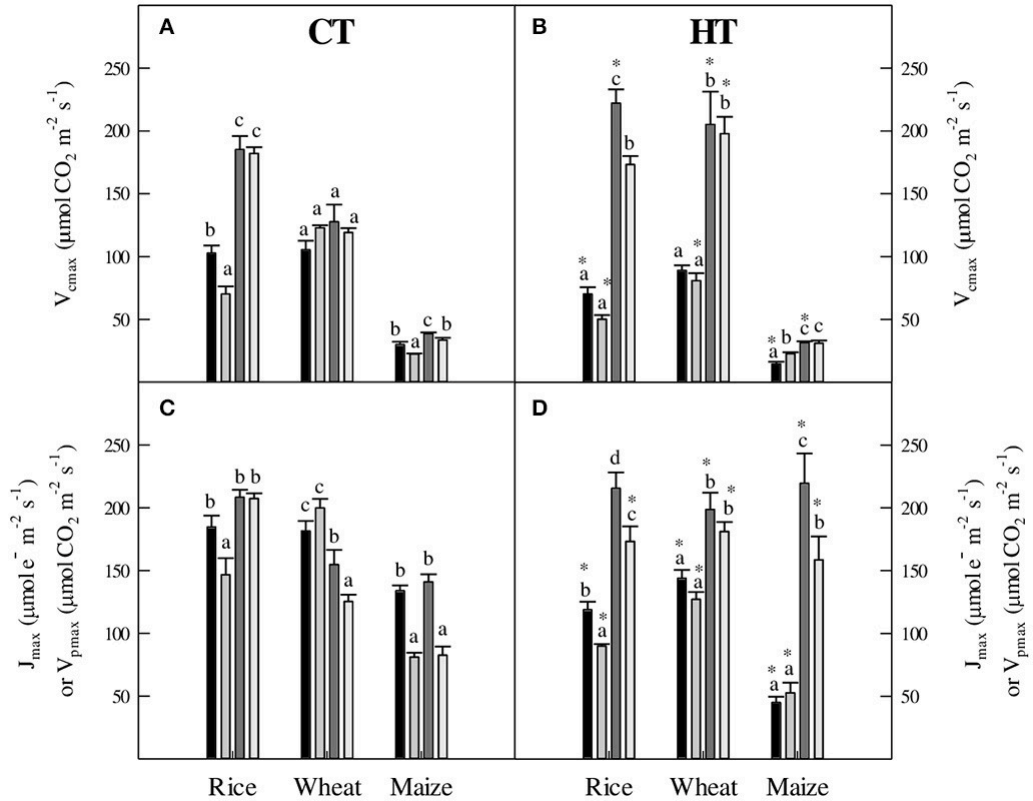
**(A,B)** The maximum velocity of Rubisco carboxylation (V_cmax_), **(C,D)** the maximum electron transport rate (J_max_) and the maximum velocity of PEPC carboxylation (V_pmax_), in plants grown at CT **(A,C)** and HT **(B,D)**. V_cmax_ were measured for wheat, rice and maize, J_max_ in wheat and rice, and V_pmax_ only in maize. All parameters were measured at 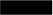 WW-25°C, 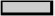 WD-25°C, 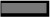 WW-38°C, and 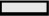 WD-38°C. Values are means ± standard error (*n* = 3–5). Different letters denote statistically significant differences among treatments within each species and same growth temperature and asterisks between the two growth temperatures within the same species, irrigation treatment and temperature of measurement (Duncan analysis *P* < 0.05).

In general, for rice and wheat, the effect of temperature was more evident than that of water availability on the shape of the A_N_-C_c_ curves (Figures S3, S4). This observation suggests a higher resilience of photosynthetic biochemistry to water deficit than to high temperatures. The biochemical parameters derived from A_N_-C_c_ curves confirmed this same pattern. In CT plants, the maximum velocity of Rubisco carboxylation (V_cmax_) was more responsive to the increase in measuring temperature from 25°C to 38°C in rice than in wheat (Figure 3A). By contrast, both species showed decreased V_cmax_ in HT plants when lowering the measuring temperature from 38°C to 25°C (Figure 3B). Significant effects of WD on V_cmax_ were observed in rice under CT-25°C and HT-38°C, and were absent in wheat.

Compared to V_cmax_, the maximum rate of electron transport (J_max_) was less affected by changes in the temperature of measurement, but similarly by changes in the irrigation treatment (Figures 3C,D). In consequence, in both rice and wheat, the ratio J_max_/V_cmax_ was lower when measured at 38°C compared to 25°C, irrespective of the growth temperature (data not shown). The effect of the growth temperature on J_max_/V_cmax_ ratio was significant when plants were measured at 38°C in the two species. By contrast, significant effects of WD were restricted to CT-25°C rice and CT-38°C wheat.

Long-term growth under WD had more evident effects on the shape of A_N_-C_s_ curves in maize compared to the effects on the A_N_-C_c_ curves in the C_3_ crops (Figure S5). However, these effects were restricted to the linear part of the A_N_-C_s_ curve, informative of PEPC activity. Accordingly, the maximum rate of PEPC carboxylation (V_pmax_) was affected by WD under all treatments except HT-25°C (Figures 3C,D). The effect of the measuring temperature on V_pmax_ was dependent on the growth temperature: no effects were observed in CT-grown plants, but V_pmax_ decreased dramatically in HT-grown plants when the measurement temperature decreased from 38°C to 25°C. V_pmax_ was also highly responsive to the growth temperature, showing a high capacity for thermal acclimation (i.e., highest values when V_pmax_ was measured at the respective growth temperature). In maize, V_cmax_ was also significantly affected by the irrigation treatment in plants grown at CT, while at HT-25°C V_cmax_ increased under WD (Figures 3A,B). Likewise, V_cmax_ in maize increased with the measuring temperature at both growth temperatures irrespective of water availability (Perdomo, 2015).

### Kinetic properties of rubisco and PEPC and their relevance for modeling photosynthesis of C_3_ and C_4_ plants

**Figure 4:**
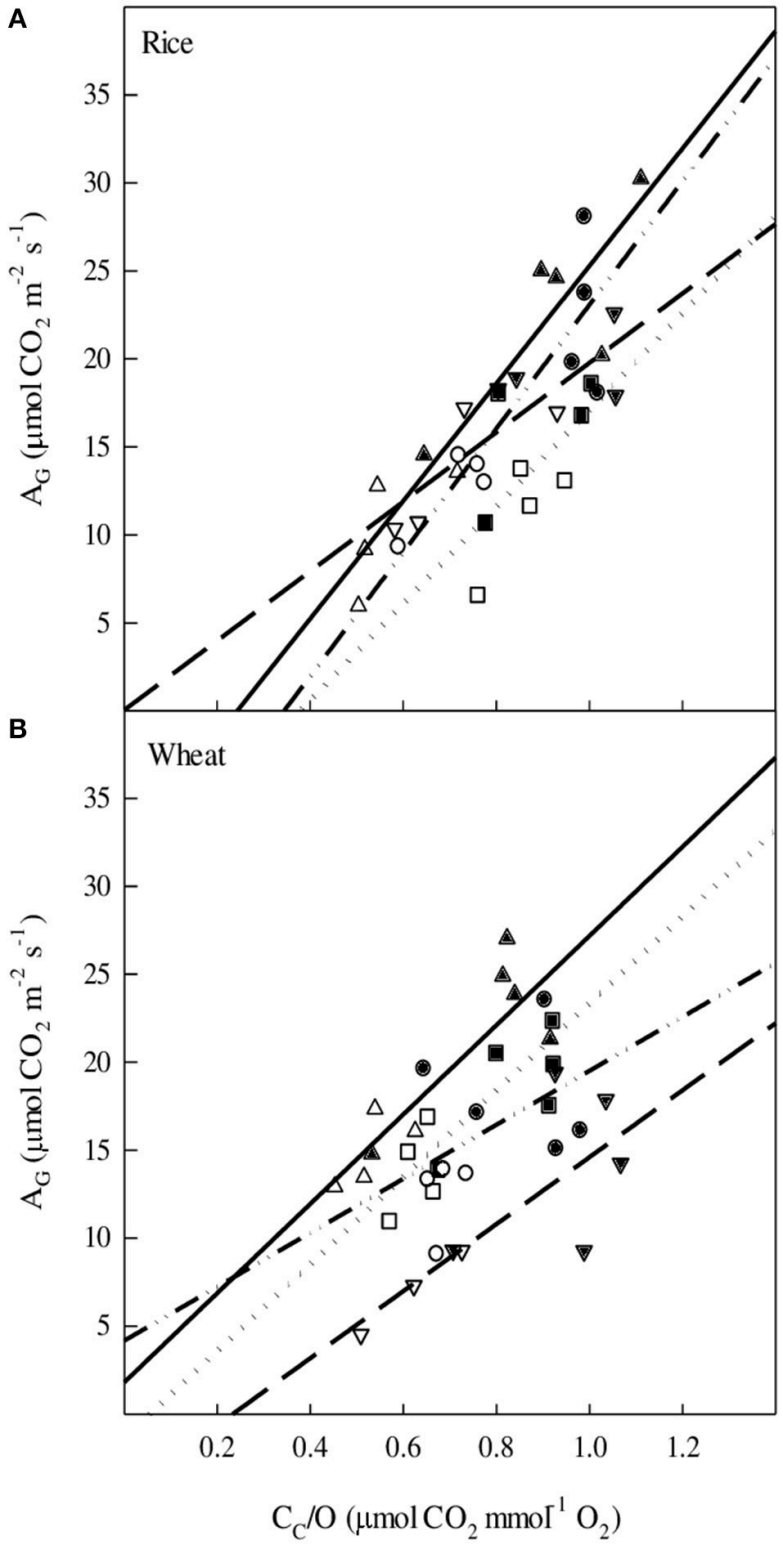
**The relationship between: the gross photosynthesis rate (A_G_) and the relative concentrations of CO_2_ and O_2_ (C_c_/O) for (A)** rice and **(B)** wheat. Symbols, treatments and lines as follows: ▴ CT-WW-25°C, ▵ CT-WD-25°C, solid regression (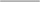); ▾ CT-WW-38°C, ▿ CT-WD-38°C, dashed regression (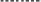); ■ HT-WW-25°C, □ HT-WD-25°C, dotted regression (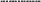); • HT-WW-38°C, ° HT-WD-38°C, dashed-dotted regression (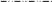). In rice, solid regression *R*^2^ = 0.90 *P* < 0.001, dashed regression *R*^2^ = 0.69 *P* < 0.01, dotted regression *R*^2^ = 0.54 *P* < 0.05, and dashed-dotted regression *R*^2^ = 0.77 *P* < 0.001. In wheat, solid regression *R*^2^ = 0.79 *P* < 0.001, dashed regression *R*^2^ = 0.60 *P* < 0.01, and dotted regression *R*^2^ = 0.65 *P* < 0.01.

The gross CO_2_ assimilation rate (A_G_) was calculated from the sum of A_N_ and half of the mitochondrial respiration in the dark (R_dark_). In rice and wheat, A_G_ increased linearly with the ratio of CO_2_ and O_2_ concentrations in the chloroplast (C_c_/O) (Figure 4). For a given temperature treatment, WD plants showed a lower A_G_ due to decreased C_c_/O, in both rice and wheat. It is remarkable that rice plants measured at the same temperature but grown at different temperatures (e.g., compare CT-25°C and HT-25°C) presented different A_G_ values for a given C_c_/O, suggesting that the carboxylase/oxygenase activity of Rubisco was sensitive to the growth temperature.

Rubisco and PEPC kinetic constants, required for photosynthesis modeling, were measured *in vitro* at the two temperatures of measurement to enable a more accurate modeling. As expected, all kinetic constants increased at 38°C with respect to 25°C in the three species (Table 1). Differences between the two C_3_ crops and maize were significant for the Michaelis–Menten constant of Rubisco for CO_2_ (K_c_) at both temperatures and for the Michaelis–Menten constant of Rubisco for O_2_ (K_o_) at 38°C.

**Table 1:** **Kinetic parameters of Rubisco and PEPC from rice, wheat, and maize measured at 25 and 38°C**.

**Species**	**K_c_ (Pa)**		**K_o_ (kPa)**		Γ^*^ (Pa)		**K_P_ (Pa)**	
	**25°C**	**38°C**	**25°C**	**38°C**	**25°C**	**38°C**	**25°C**	**38°C**
Rice	29.1 ± 1.6^a^	87.7 ± 5.2^a^^*^	45.7 ± 4.6^a^	58.3 ± 5.7^a^^*^	4.19 ± 0.19^a^	6.34 ± 0.36^b^^*^	–	–
Wheat	31.6 ± 1.1^a^	89.3 ± 3.6^a^^*^	39.2 ± 3.8^a^	50.3 ± 4.5^a^^*^	3.87 ± 0.21^a^	5.32 ± 0.32^a^^*^	–	–
Maize	85.8 ± 7.0^b^	188.3 ± 5.7^b^^*^	39.8 ± 3.5^a^	74.5 ± 7.5^b^^*^	4.30 ± 0.30^a^	6.47 ± 0.26^b^^*^	8.0 ± 3.0	13.2 ± 5.0^*^

Four replicates were used for the Michaelis–Menten constants of Rubisco for CO_2_ and O_2_ (K_c_ and K_o_) and the Michaelis–Menten constant of PEPC for CO_2_ (K_P_), and five replicates for the Rubisco specificity factor (S_c/o_). The chloroplast CO_2_ compensation point in the absence of mitochondrial respiration (Γ^*^) was calculated from S_c/o_ as explained in the Material and Methods. Different letters and asterisks denote statistically significant differences (Duncan analysis, P < 0.05) among species within the same temperature and between measurement temperatures within the same species, respectively.

The V_cmax_ estimated for the C_3_ species by applying the model of Farquhar et al. (1980), and using the values of the *in vitro* Rubisco kinetics specific for each species at each measurement temperature/VPD (Table 1) tended to be lower than the V_cmax_ estimated using the kinetic parameters from Bernacchi et al. (2001, 2002) (Table 2). However, the estimates obtained by each method for the different species under each treatment were highly related. None of the differences between V_cmax_ values estimated using specific kinetics and kinetics from Bernacchi et al. (2001, 2002) were significant in rice, and only in 3 cases the differences were significant in wheat (CT-WD-25°C, HT-WW-25°C and HT-WD-25°C). Indeed, the correlation between the two V_cmax_ estimates was high (*r*^2^ > 0.99) in both species, however the slope in wheat (0.86) was lower than in rice (0.98) and thus more distant to a 1:1 relationship (data not shown). Significant differences between values of J_max_ estimated using specific and Bernacchi kinetics (Bernacchi et al., 2001, 2002) were observed for three treatments in rice (CT-WW-25°C, CT-WD-25°C, and HT-WD-25°C) and two in wheat (CT-WW-25°C and CT-WD-25°C) (Table 2). To strengthen these observations, the estimates for V_cmax_ and J_max_ obtained using the species-specific values of Rubisco kinetics measured in the present study and those reported for tobacco in Bernacchi et al. (2001, 2002) were compared by applying the method described by Ethier and Livingston (2004); significant differences in V_cmax_ were observed in two cases for each species, while significant differences in J_max_ were only observed in CT-WD-25°C wheat (Table S1).

**Table 2:** **Comparison of the maximum velocity of Rubisco carboxylation (V_cmax_) and maximum rate of electron transport (J_max_) in plants grown at CT and HT, under WW and WD, and measured at 25 and 38°C, using the Rubisco kinetics parameters (K_c_, K_o_, and S_c/o_) measured in the present study for rice and wheat (own kinetics), and using the parameters reported for tobacco by Bernacchi et al. (2001, 2002)**.

**Species**	**Growth T (°C)**	**Irrigation treatment**	**Measurement T (°C)**	**Own kinetics**		**Kinetics by Bernacchi et al. (2001, 2002)**	
				**V_cmax_**	**J_max_**	**V_cmax_**	**J_max_**
Rice	CT	WW	25	102.7±6.1	184.5±9.2	116.0±6.0	112.7±22.3^*^
Rice	CT	WD	25	70.1±6.2	146.7±13.2	77.9±7.2	82.8±4.5^*^
Rice	CT	WW	38	185.1±10.8	208.3±5.9	206.2±10.8	197.6±6.9
Rice	CT	WD	38	182.2±5.0	207.3±4.2	195.8±3.1	198.5±0.3
Rice	HT	WW	25	70.2±5.4	118.8±6.6	78.0±5.8	108.8±5.1
Rice	HT	WD	25	50.2±3.3	90.0±1.6	59.2±3.8	79.3±2.7^*^
Rice	HT	WW	38	222.1±11.0	215.4±12.6	228.3±14.4	198.5±11.0
Rice	HT	WD	38	173.2±6.8	173.4±11.8	176.5±15.2	161.4±5.8
Wheat	CT	WW	25	105.4±7.2	181.5±7.9	125.8±9.7	101.5±15.4^*^
Wheat	CT	WD	25	122.9±2.1	199.8±7.1	144.1±3.3^*^	81.9±2.5^*^
Wheat	CT	WW	38	127.7±13.7	154.7±11.6	151.6±16.2	156.9±11.3
Wheat	CT	WD	38	119.2±3.3	125.3±5.4	134.5±8.9	119.7±5.8
Wheat	HT	WW	25	88.9±4.2	143.8±6.8	110.0±6.1^*^	133.6±5.9
Wheat	HT	WD	25	80.9±5.9	127.2±5.7	104.5±6.1^*^	122.8±6.6
Wheat	HT	WW	38	205.2±26.0	198.7±13.3	247.7±29.2	186.8±10.9
Wheat	HT	WD	38	197.8±13.5	181.1±7.6	230.3±15.9	174.2±8.6

V_cmax_ and J_max_ were calculated on a C_c_ basis as estimated by the method of Harley et al. (1992). Values are means ± standard errors (n = 5). Asterisks denote statistically significant differences between the two V_cmax_ or J_max_ values (Duncan analysis, P < 0.05) within the same treatment (growth T and irrigation treatment).

Regarding C_4_ modeling, the comparison was established between V_cmax_ and V_pmax_ estimates using the Rubisco and PEPC kinetics reported in the present study for maize and those reported in von Caemmerer (2000) (Table 3). von Caemmerer (2000) used the temperature dependence of Rubisco kinetic constants reported by Bernacchi et al. (2002), while K_p_ was assumed to be invariable with temperature. Differences in V_cmax_ estimates between the two approaches were non-significant under all treatments, while significant differences in V_pmax_ were found in CT-grown plants, irrespective of the irrigation treatment and the measuring temperature (Table 3).

**Table 3:** **Comparison of the maximum velocity of Rubisco carboxylation (V_cmax_) and the maximum velocity of PEPC carboxylation (V_pmax_) in plants grown at CT and HT, under WW and WD, and measured at 25 and 38°C, using Rubisco and PEPC kinetic parameters (K_c_, K_*o*_, S_c/o_, and K_p_) measured in the present study for maize, and using the parameter values reported by von Caemmerer (2000)**.

**Species**	**Growth T (°C)**	**Irrigation treatment**	**Measurement T (°C)**	**Own kinetics**		**Kinetics by von Caemmerer (2000)**	
				**V_cmax_**	**V_pmax_**	**V_cmax_**	**V_pmax_**
Maize	CT	WW	25	29.9±2.3	133.8±4.3	29.7±2.2	112.8±3.8^*^
Maize	CT	WD	25	22.5±0.3	81.1±3.5	22.3±0.3	69.5±2.6^*^
Maize	CT	WW	38	38.7±1.0	141.1±5.9	41.5±0.9	117.5±6.4^*^
Maize	CT	WD	38	33.6±1.7	82.6±6.9	37.5±0.8	62.0±4.4^*^
Maize	HT	WW	25	14.4±1.8	44.9±4.7	14.1±1.8	38.4±4.1
Maize	HT	WD	25	22.7±1.2	52.8±8.2	22.2±1.2	46.8±7.0
Maize	HT	WW	38	31.7±0.9	219.5±23.7	32.6±1.1	182.0±15.0
Maize	HT	WD	38	31.1±2.2	158.7±18.6	32.2±2.2	141.4±10.0

Values are means ± standard errors (n = 5). Asterisks denote statistically significant differences between the two V_cmax_ or V_pmax_ values (Duncan analysis, P < 0.05) within the same treatment (growth T and irrigation treatment).

### Sensitivity and acclimation capacity to high temperature and water deficit in rice, wheat, and maize

A temperature sensitivity index (TSI, Table 4) was calculated for the main photosynthetic parameters as the ratio between the value at CT-25°C and that at CT-38°C. The photosynthetic machinery of maize was particularly insensitive to the sudden increase in the measuring temperature in CT-grown plants, both under WW and WD. By contrast, A_N_, g_s_, and g_m_ were affected by short-term heat stress in rice and wheat (as denoted by the asterisks), although relative sensitivity was dependent on the irrigation treatment. Irrespective of the irrigation treatment, wheat was the unique species with TSI > 1 for J_max_, and rice presented the lowest TSI for V_cmax_ (i.e., the largest increment due to the increase in the temperature of measurement). R_dark_ was the most sensitive parameter to the increase in the temperature of measurement, particularly under WW conditions.

**Table 4:** **Temperature sensitivity index (TSI) for the net CO_2_ assimilation rate (A_N_), stomatal conductance (g_s_), mesophyll conductance (g_m_), maximum velocity of Rubisco carboxylation (V_cmax_), maximum rate of electron transport (J_max_), maximum velocity of PEPC carboxylation (V_pmax_), and mitochondrial respiration at pre-dawn (R_dark_)**.

**Parameter**	**WW**			**WD**		
	**Rice**	**Wheat**	**Maize**	**Rice**	**Wheat**	**Maize**
A_N_	1.6±0.2^*^	1.7±0.2^*^	0.9±0.1	0.8±0.2	2.0±0.8^*^	0.8±0.2
g_s_	1.5±0.3	1.0±0.2	0.7±0.1	0.8±0.3	1.5±0.3^*^	0.7±0.2
g_m_	2.5±0.9^*^	1.7±0.2^*^	–	1.0±0.4	4.4±1.3^*^	–
V_cmax_	0.6±0.1^*^	0.9±0.1	0.8±0.1	0.4±0.1^*^	1.0±0.1	0.7±0.1^*^
J_max_	0.9±0.1	1.2±0.1	–	0.7±0.1^*^	1.6±0.1^*^	–
V_pmax_	–	–	1.0±0.1	–	–	1.0±0.1
R_dark_	0.2±0.1^*^	0.2±0.1^*^	0.1±0.1^*^	0.3±0.1^*^	0.3±0.1^*^	0.2±0.1^*^

TSI was calculated, under both well-watered (WW) and water deficit (WD) conditions, as the ratio between the values from plants grown and measured at 25°C and those from plants grown at CT and measured at 38°C [TSI = (CT-25°C)/(CT-38°C)]. The asterisks indicate significant differences between CT-25°C and CT-38°C. Values are means ± standard errors (n = 5).

The temperature acclimation index (TAI, Table 5) provides a tool for comparison of plants grown and measured at the same temperature (CT-25°C and HT-38°C). Under WW, wheat was the species with the lowest TAI for A_N_, and both rice and wheat presented TAI > 1 for V_cmax_, while maize TAI for V_pmax_ was also >1 (Table 5). Under WD, maize was the unique species with TAI for A_N_ significantly higher than 1, all three species presented TAI > 1 for g_s_ and V_cmax_, and maize for V_pmax_. TAI for R_dark_ was not significantly different from 1 under WD but increased under WW conditions in all species. In wheat WW, TAI <1 for A_N_ and TAI >1 for R_dark_, suggesting a lower capacity of acclimation to increased temperature (Perdomo, 2015).

**Table 5:** **Temperature acclimation index (TAI) for the net CO_2_ assimilation rate (A_N_), stomatal conductance (g_s_), mesophyll conductance (g_m_), maximum velocity of Rubisco carboxylation (V_cmax_), maximum rate of electron transport (J_max_), maximum velocity of PEPC carboxylation (V_pmax_), and mitochondrial respiration at pre-dawn (R_dark_)**.

**Parameter**	**WW**			**WD**		
	**Rice**	**Wheat**	**Maize**	**Rice**	**Wheat**	**Maize**
A_N_	0.9±0.1	0.7±0.1^*^	1.0±0.1	1.0±0.4	0.9±0.1	1.3±0.1^*^
g_s_	1.2±0.1	0.8±0.1	1.1±0.1	1.6±0.6	2.4±0.5^*^	1.3±0.1^*^
g_m_	0.6±0.1	0.8±0.1	–	0.8±0.5	0.2±0.1^*^	–
V_cmax_	2.2±0.1^*^	1.9±0.2^*^	1.1±0.1	2.6±0.3^*^	1.6±0.1^*^	1.4±0.1^*^
J_max_	1.2±0.1	1.1±0.1	–	1.2±0.2	0.9±0.1	–
V_pmax_	–	–	1.6±0.2^*^	–	–	2.0±0.4^*^
R_dark_	2.1±1.0^*^	5.5±1.3^*^	2.3±0.3^*^	0.8±0.1	0.8±0.2	1.1±0.2

TAI was calculated, under both well-watered (WW) and water deficit (WD) conditions, as the ratio between the values from plants grown and measured at 38°C and those from plants grown and measured at 25°C [TAI = (HT-38°C)/(CT-25°C)]. The asterisks indicate significant differences between CT-25°C and HT-38°C. Values are means ± standard errors (n = 5).

## Discussion

Long-term responses to increased temperature, VPD and drought stress were compared in rice, wheat, and maize, to improve our understanding of how these three major global crops will respond to the future climate. In addition, we used the data obtained as well as the Rubisco *in vitro* kinetics for each species and treatments to check the validity of commonly employed “universal Rubisco constants” to parameterize photosynthesis models in different species. These two objectives are discussed independently in the next sections.

### Long-term acclimation to high temperature and drought stress in three important global crops

Plants grown and measured at 25°C under well-watered conditions (CT-WW-25°C) showed similar values for A_N_ in the three species (Figure 1). However, WD resulted in a significant decrease of A_N_ in all three species, the strongest effect being observed in rice and the mildest in maize. In the two C_3_ species, these limitations were mostly due to stomatal conductance (g_s_), which largely decreased in both species, while the mesophyll conductance to CO_2_ (g_m_) decreased under water deficit in rice but apparently increased in wheat (Kalaji and Nalborczyk, 1991; Choluj et al., 1997). Parameters reflecting photosynthetic activity (V_cmax_ and J_max_) were largely unaffected by WD (Figure 3), as is often found in C_3_ species (Flexas et al., 2002, 2006b; Galmés et al., 2007b). In C_4_ maize, the drought stress-induced decrease in A_N_ was due to combined decreases on V_cmax_ and V_pmax_ plus g_s_ only when measured at 38°C (Figures 1, 3); this is in agreement with previous reports in C_4_ plants (Lal and Edwards, 1996; Carmo-Silva et al., 2010).

When WW plants grown at CT were measured at 38°C, significant decreases of photosynthesis were observed in all species but maize, although these effects were of smaller magnitude than those induced by WD except in wheat, where the two stresses resulted in responses of similar magnitude (Figure 1). These results suggest that, of the three crops, rice was the most sensitive species to drought stress and wheat was the most sensitive to increased measurement temperature, while maize was the least sensitive to both drought stress and increased measurement temperature (see also Table 4). In many studies, short-term responses are taken as evidence to predict the future photosynthetic performance of a given species under a changing climate. However, there are at least two factors that can bias these responses: (i) interactions between stresses, and (ii) acclimation in the long-term (Centritto et al., 2002; Flexas et al., 2006a; Vile et al., 2012; Cheesman and Winter, 2013). Regarding interactions, these are evidenced by measuring at 38°C plants grown at CT and subjected to water deficit (CT-WD-38°C). In rice, A_*N*_ values were somewhat larger under WD when plants were measured at 38°C than at 25°C, however, the effect of WD at 38°C was not significant (Figure 1). In other words, in rice the high measurement temperature and drought stress interacted somehow to increase photosynthesis as compared to measuring drought plants at the lower temperature. In wheat, in contrast, the interaction of high measurement temperature and WD resulted in a cumulative effect of both stresses, so that photosynthesis under WD-38°C was about half of the value observed when stress treatments were applied independently (Figure 1).

Considering the comparison of WW-CT plants measured at 25°C and WD-CT plants measured at 38°C, it can no longer be considered that rice is more drought stress sensitive and wheat more temperature sensitive. Instead, both species are similarly sensitive to the combination of high measurement temperature and drought stress. This result illustrates how short-term studies observing the response to isolated stresses may fail to reproduce plant responses to the most complex, combined stress conditions that are often experienced in the field (Shah and Paulsen, 2003; Prasad et al., 2008; Vile et al., 2012; Tozzi et al., 2013). In maize, the combination of high temperature/VPD and WD resulted in photosynthesis rates only marginally lower than those displayed by WW plants measured at low temperature, as expected for a C_4_ species (Edwards et al., 2001; Crafts-Brandner and Salvucci, 2002; Osborne and Beerling, 2006).

Long-term acclimation responses may further confound the predictive value of short-term observations. Acclimation was evident for the three species. Values of A_N_ were very similar between plants grown and measured at 25°C and those grown and measured at 38°C (i.e., TAI close to 1, Table 5), both under WW and WD conditions (Figures 1A,B). Only in WW wheat A_N_ was lower in plants grown and measured at high temperature (HT-WW-38°C) than in plants grown at control temperature and measured at 25°C (CT-WW-25°C), and in WD maize A_N_ was higher in plants grown and measured at 38°C than at 25°C, confirming the adaptation of these two species to cool and hot temperature conditions, respectively (Hikosaka et al., 2006; Nagai and Makino, 2009; Yamori et al., 2009). A similar acclimation to growth temperature—i.e., A_N_ is kept constant—has also been observed in poplar (Silim et al., 2010). Interestingly, acclimation of photosynthesis to high temperature in the three species was achieved through different homeostatic mechanisms. For instance, in both WW and WD rice and WD wheat, the same A_N_ at the two temperatures was achieved by increasing g_s_ and V_cmax_ but decreasing g_m_ (Figures 1, 3). Contrarily, in WW wheat, a lower A_N_ was observed at high temperature despite increased V_cmax_, which was in part attributable to large increases in respiration (Figure 2). These results indicate that changing climate results in species-dependent changes in the ratios between biochemical and diffusive parameters even in cases where net photosynthesis does not change.

In summary, the present results illustrate that the photosynthetic responses to climate conditions—e.g., drought stress and increased temperature and VPD—differ when analyzed in the short- or long-term, in a species-specific manner. Therefore, it is necessary to be cautious when deriving generalizations or predictions from short-term studies with few species subjected to isolated stress conditions. Rather, detailed long-term experiments with different species and stress interactions are urged for a better understanding of crop responses to withstanding climate change conditions.

### Species-specific rubisco kinetics and their effects on accurate parameterization of C_3_ and C_4_ photosynthesis models

Photosynthesis models such as that of Farquhar et al. (1980) for C_3_ plants, or that of von Caemmerer (2000) for C_4_ plants, allow the estimation of biochemical traits such as the maximum velocity of Rubisco carboxylation (V_cmax_) and the maximum rate of electron transport (J_max_) in C_3_ plants, and the maximum velocities of PEPC (V_pmax_) and Rubisco (V_cmax_) carboxylation in C_4_ species (von Caemmerer, 2013). While this parameterization was originally applied on a C_*i*_ basis (Berry and Björkman, 1980; Farquhar et al., 1980), it is now widely recognized that correct parameterization should take into account the CO_2_ concentration at the Rubisco site inside chloroplasts (C_c_), for which knowledge of the mesophyll conductance to CO_2_ (g_m_) is required. For instance, a recent survey on 130 species reveals that assuming infinite g_m_ underestimates, on average, V_cmax_ by as much as 75% and J_max_ by 60% (Gu and Sun, 2014; Sun et al., 2014). Under severe drought stress conditions the underestimations may be even larger (Flexas et al., 2006b).

On the other hand, g_m_ typically decreases under drought stress (Flexas et al., 2002; Galmés et al., 2007a; Gallé et al., 2009; Hu et al., 2010) and increases with temperature, at least up to a certain threshold (Silim et al., 2010; Evans and von Caemmerer, 2013; Walker et al., 2013). The response of g_m_ to temperature has recently been shown to be strongly species-dependent (von Caemmerer and Evans, 2015), although the mechanisms for this are still unclear (Flexas and Díaz-Espejo, 2015; von Caemmerer and Evans, 2015). Therefore, to correctly parameterize photosynthesis, g_m_ should be precisely determined for plants at each experimental condition and measurement temperature. In this study, g_m_ decreased in rice after the increase in the measurement temperature, but this decrease was not significant in wheat. In contrast, an increase in g_m_ with measurement temperature was reported by von Caemmerer and Evans (2015) and supported by data from Xiong et al. (2015) in rice. A recent reported showed a decline in g_m_ as leaves aged from fully expanded to senescing (Barbour et al., 2016), supporting that the above discrepancies reflect the importance of the experimental conditions and leaf age.

Several problems with existing methods for the estimation of g_m_ have been raised recently (Tholen et al., 2012; Gu and Sun, 2014). On one hand, the estimated g_m_ may not reflect purely a diffusion conductance, because A_N_ reflects a CO_2_ net flux that combines photosynthesis, photorespiration and mitochondrial respiration, and these three processes move CO_2_ along different distances and diffusion pathways (Tholen et al., 2012). On the other hand, apparent responses of g_m_ to varying light and CO_2_ may be artefactual, resulting from analysis of g_m_ dependence on variables that are explicitly included in the equations used to calculate g_m_ (Gu and Sun, 2014). These type of errors should affect only methods that estimate different g_m_ values at any given C_i_ (i.e., Harley et al., 1992 and Yin et al., 2009), but not methods that solve for a single g_m_ estimate along a C_i_ gradient (i.e., Ethier and Livingston, 2004). Since, in most cases, the estimates based on these three different methods of estimation show a significant agreement (Figure S1) we may dismiss the importance of these errors in the present study, but since different values were obtained with the different methods for some treatments potential errors cannot be completely ruled out.

Hence, while recognizing that some of the values presented may represent an approximation to the *true* g_m_, we can still use these predictions to check for the effects of species-specific differences in Rubisco kinetic constants and their temperature response and acclimation on the parameterization of photosynthesis models. This is because in addition to precise knowledge on g_m_ and its temperature dependency, *a priori* knowledge of Rubisco kinetic constants (S_c/o_, K_c_, and K_o_), as well as their temperature dependencies, is required to parameterize photosynthesis models. Since these constants are unknown for Rubiscos from many species, it is becoming a common practice to use “standard” constants for any given species. The most commonly used “standard” Rubisco kinetics and temperature functions are those for tobacco as obtained by Bernacchi et al. (2002). However, it is well documented that significant differences occur among species in Rubisco kinetics (e.g., Galmés et al., 2005, 2015; Savir et al., 2010; Orr et al., 2016; Prins et al., 2016), and these differences result in significant bias in model parameterization (Walker et al., 2013). These authors also indicate that *in vitro* Rubisco kinetics may not accurately describe the operation of Rubisco under physiological conditions, due to degradation and/or inactivation of the enzyme during extraction or differences in the *in vitro* assay conditions compared to the chloroplast stroma. While the latter may be true, degradation, or activation of Rubisco during the extraction may affect quantitatively absolute parameters, such as the maximum Rubisco activity or Rubisco concentration, but should not affect S_c/o_, K_c_, and K_o_.

Determination of *in vivo* kinetics for a large number of species with different functional types, as urged by Walker et al. (2013), may not be accomplished in the short term, as such experiments require the use of mutants with low Rubisco contents for each species, growth under low CO_2_ concentrations and the use of gas exchange measurements at different oxygen partial pressures, i.e., plant material that is yet to be created and techniques that are not readily available except in very few laboratories. In contrast, measuring *in vitro* kinetic constants of Rubisco is easier and less time consuming, so that a number of different species can be characterized in a reasonable time (Hermida-Carrera et al., 2016; Orr et al., 2016; Prins et al., 2016). Therefore, we propose using Γ^*^ derived from *in vitro* S_c/o_ measured in each species at different temperatures to first estimate g_m_ and, then, parameterize photosynthesis from A_N_-C_c_ curves using the species and temperature specific *in vitro* kinetics of Rubisco rather than “standard” values determined for model species.

The *in vitro* values for Γ^*^ and K_c_ are within the range of values obtained *in vivo* for tobacco and Arabidopsis by Walker et al. (2013), supporting the use of *in vitro* values as a valid approach to estimate Rubisco constants comparable to those operating *in vivo*. Nevertheless, K_o_ was almost the double in rice and wheat regarding the tobacco and Arabidopsis also reported by Walker et al. (2013), which demonstrates the differences in the Rubisco kinetic parameters among species and point out the importance of considering the species-specific values instead of general “consensus values.”

Rubisco from C_4_ maize had a lower affinity for CO_2_ (i.e., higher K_c_) than the Rubisco from C_3_ rice and wheat (Table 1), in agreement with previous reports (Christin et al., 2008; Carmo-Silva et al., 2010; Cousins et al., 2010; Savir et al., 2010; Whitney et al., 2011). At 25°C, differences in Γ^*^ and K_o_ between species and photosynthetic mechanisms were non-significant, indicative that maize Rubisco presents a higher maximum catalytic turnover for the carboxylation reaction (k_cat_^c^) than the two C_3_ species, which is in agreement with recent studies (Sharwood et al., 2016a,b). The response of Rubisco kinetics to increased temperature followed trends already described in the literature, both *in vivo* (Brooks and Farquhar, 1985; Bernacchi et al., 2001; Walker et al., 2013) and *in vitro* in C_3_ and C_4_ species (Badger and Collatz, 1977; Jordan and Ogren, 1984; Galmés et al., 2005; Boyd et al., 2015), with increases in K_c_, K_o_, K_c_/K_o_, and Γ^*^ (Table 1). The relative increase in Γ^*^ with temperature was lower in wheat than in maize and rice. There are few reported measurements of the Michaelis–Menten constant for PEPC (K_p_) in C_4_ species (Bauwe, 1986; Pfeffer and Peisker, 1998; Boyd et al., 2015) and limited studies on the temperature dependence of K_p_ (Boyd et al., 2015). K_p_ increased with temperature in *Setaria viridis* (Boyd et al., 2015) and in maize (this study) to a lesser extent than the increase of K_c_. This fact, together with the higher temperature-driven increase of V_pmax_ as compared to that of V_cmax_ (Figure 3), suggests increased Rubisco limitations for C_4_ photosynthesis at high temperatures. That the K_p_ values for *S. viridis* are higher than those reported here for maize, corroborates the need to use species-specific kinetic constants for both Rubisco and PEPC for greater accuracy in C_4_ photosynthetic modeling.

Using each species Rubisco constants resulted in model parameterization estimates that in some cases differed significantly from those obtained using the “standard” constants by Bernacchi et al. (2002) in C_3_ plants. These differences represented on average 10–20% overestimation of V_cmax_ and a largely variable underestimation of J_max_ (Table 2), with strongly biased V_cmax_/J_max_ ratios. The magnitude of these discrepancies, similar to that found by Walker et al. (2013), is remarkable, especially considering that the species compared (tobacco and Arabidopsis in Walker's study, rice and wheat here) are all herbaceous angiosperms. It is likely that even broader deviations would occur when using “standard” tobacco kinetics to parameterize more distant species, like woody angiosperms, gymnosperms, ferns or mosses. Part of this bias in the parameterization of V_cmax_ and J_max_ is due to bias in the estimation of g_m_, as indicated by the significant differences obtained between the g_m_ values estimated using the Rubisco kinetic values from the present study and those reported in Bernacchi et al. (2001, 2002). These differences were observed regardless of the g_m_ estimation method (i.e., Harley et al., 1992; Ethier and Livingston, 2004 or Yin et al., 2009; Table S2). These results also demonstrate that species- and temperature-specific kinetic parameters for PEPC and Rubisco are required for accurate photosynthesis parameterization in C_4_ plants, in particular for estimating V_pmax_ (Table 3).

In summary, the present results confirm and extend the conclusion by Walker et al. (2013) that species-specific differences in *in vivo* Rubisco parameters are large enough to significantly bias modeling of C_3_ photosynthesis. It is further shown, for the first time, that differences in species-specific kinetics are large enough to bias modeling of C_4_ photosynthesis. It is thus strongly recommended that the use of “standard” Rubisco kinetics from tobacco is avoided when modeling photosynthesis in other species. As obtaining *in vivo* Rubisco kinetics for different species is not achievable in the short-term, we propose to use *in vitro* kinetics as determined by the methods explained here and elsewhere (Kane et al., 1994; Ruuska et al., 1998; Parry et al., 2007; Shay and Kubien, 2013; Perdomo, 2015; Galmés et al., 2016; Orr et al., 2016; Prins et al., 2016) as a first proxy for *in vivo* kinetics.

## Author contributions

JAP performed the experiment, analyzed the data, and wrote the paper. ECS contributed to the design, analysis, and interpretation of the C_4_ gas-exchange model data. CH was involved in the acquisition of the Rubisco kinetic data. JF is an expert in plant physiology, contributed substantially to write the paper, and critically revised the work. JG obtained funding for the project and was a substantial contributor to the conception and design of the work.

## Funding

This study was financially supported by the contract AGL2009-07999 (Plan Nacional, Spain) awarded to JG. JAP was the recipient of a FPI grant from the Govern de les Illes Balears. ECS was the recipient of a Rothamsted Research Career Fellowship that currently supports JAP. Rothamsted Research receives grant-aided support from the Biotechnology and Biological Sciences Research Council (BBSRC) 20:20 Wheat® Institute Strategic Programme.

### Conflict of interest statement

The authors declare that the research was conducted in the absence of any commercial or financial relationships that could be construed as a potential conflict of interest.

## Abbreviations

A_G_: gross CO_2_ assimilation rate
A_N_: net CO_2_ assimilation rate
C_a_: atmospheric CO_2_ concentration
C_c_: chloroplastic CO_2_ concentration
C_i_: intercellular CO_2_ concentration
CT: control temperature
F_o_: basal fluorescence of a dark adapted leaf
F_s_: steady-state fluorescence signal
g_bs_: bundle sheath conductance to CO_2_
g_m_: mesophyll conductance to CO_2_
g_s_: stomatal conductance to CO_2_
HT: high temperature
J_max_: maximum photosynthetic electron transport rate
K_*c*_: Michaelis–Menten constant of Rubisco for CO_2_
k_cat_^c^: reaction turnover rate for carboxylation activity of Rubisco
K_o_: Michaelis–Menten constant of Rubisco for O_2_
K_P_: Michaelis–Menten constant of phosphoenolpyruvate carboxylase for HCO_3_
R_dark_: mitochondrial respiration at pre-dawn
R_L_: non-photorespiratory CO_2_ evolution in the light
R_m_: mesophyll mitochondrial respiration
PAR: photosynthetic active radiation
PSII: photosystem II
S_c/o_: *in vitro* Rubisco specificity factor for CO_2_/O_2_
TAI: temperature acclimation index
TSI: temperature sensitivity index
V_cmax_: maximum velocity of Rubisco carboxylation activity
V_pmax_: maximum velocity of PEPC carboxylation activity
VPD: vapor pressure deficit
WD: water deficit
WW: well-watered
Γ^*^: photorespiratory CO_2_ compensation point.
