# Supplementary material for: Acclimation of Biochemical and Diffusive Components of Photosynthesis in Rice, Wheat, and Maize to Heat and Water Deficit: Implications for Modeling Photosynthesis
Source: Front Plant Sci. 2016 Nov 22;7:1719. doi: 10.3389/fpls.2016.01719 (PMC5118457; doi:10.3389/fpls.2016.01719)
Supplement: Supplementary file 1 [file Table1.DOCX]

***Supplementary Material***

**Acclimation of biochemical and diffusive components of photosynthesis in rice, wheat and maize to heat and water deficit: implications for modeling photosynthesis.**

**Authors:** Juan Alejandro Perdomo^1,3^, Elizabete Carmo-Silva^2^, Carmen Hermida-Carrera^3^, Jaume Flexas^3^, Jeroni Galmés^3*^.

**Institution addresses:**

^1^Plant Biology and Crop Science, Rothamsted Research, Harpenden, UK.

^2^Lancaster Environment Centre, Lancaster University, Lancaster, UK.

^3^Research Group on Plant Biology under Mediterranean Conditions, Universitat de les Illes Balears, Balearic Islands, Spain.

**^*^Corresponding author:** Jeroni Galmés

e-mail: jeroni.galmes@uib.cat

fax: +34 971 173184

tel: +34 971 252710

**Figure S1.** Comparison of the mesophyll conductance (g_m_) estimated from three different methods: Harley et al. (1992), Ethier and Livingston (2004), Yin et al. (2009), in rice and wheat plants grown at CT and HT, under WW and WD conditions, and measured at 25ºC and 38ºC. The values for the Rubisco kinetic parameters required in the three methods were those measured *in vitro* in the present study for rice and wheat at the two temperatures, 25ºC and 38ºC (Table 1). Ethier method is based on gas exchange measurements, while Harley and Yin methods are based on combination of gas exchange and chlorophyll fluorescence measurements. Yin and Harley methods differ in that Yin includes the possible contributions of cyclic electron transport, pseudocyclic electron transport, and variable Q-cycle to balance H^+^ and e^–^ supply. Differences among methods are described in more detail by Pons *et al.* (2009). Symbols and treatments as follows: CT-WW-25ºC, CT-WD-25ºC, CT-WW-38ºC, CT-WD-38ºC, HT-WW-25ºC, HT-WD-25ºC, HT-WW-38ºC, HT-WD-38ºC.

**Figure S2.** The relationship between the net CO_2_ assimilation rate (A_N_) and the total leaf conductance (g_t_) (a, b) in rice and wheat, and the stomatal conductance (c) in maize. Symbols and treatments follows: CT-WW-25ºC, CT-WD-25ºC,CT-WW-38ºC CT-WD-38ºC, HT-WW-25ºC, HT-WD-25ºC, HT-WW-38ºC, HT-WD-38ºC. Lines and treatments as follows: solid regression () CT-25ºC, dashed regression () CT-38ºC, dotted regression () HT-25ºC, dashed-dotted regression () HT-38ºC.

**Figure S3.** Relationship between the net CO_2_ assimilation rate (A_N_) and the chloroplastic CO_2_ concentration (C_c_) in rice. Values are means ± standard error (n = 5). Axis scales have been adjusted to allow comparison among panels within this figure and with Figs. S4 & S5.

**Figure S4.** Relationship between the net CO_2_ assimilation rate (A_N_) and the chloropastic CO_2_ concentration (C_c_) in wheat. Values are means ± standard error (n = 5). Axis scales have been adjusted to allow comparison among panels within this figure and with Figs. S3 & S5.

**Figure S5.** Relationship between the net CO_2_ assimilation rate (A_N_) and the bundle sheath CO_2_ concentration (C_s_) in maize. Values are means ± standard error (n = 5). Y-axis scale has been adjusted to allow comparison among panels within this figure and with Figs. S3 & S4.

**Table S1**. Comparison of the maximum velocity of Rubisco carboxylation (V_cmax_) and maximum rate of electron transport (J_max_), estimated with the method described by Ethier and Livingston (2004), in plants grown at CT and HT, under WW and WD, and measured at 25ºC and 38ºC, using the Rubisco kinetics parameters (K_c_, K_o_ and S_c/o_) measured in the present study for rice and wheat (own kinetics), with regard to the parameters described in Bernacchi et al. (2001, 2002). Values are means ± standard errors (n = 5). Asterisks denote statistically significant differences by Duncan analysis (*P* < 0.05) between the two methods within the same treatment.

| **Species** | **Growth T**  **(ºC)** | **Irrigation Treatment** | **Measurement T**  **(ºC)** | **Own kinetics** | | **Kinetics by Bernacchi**  **et al. (2001, 2002)** | |
| --- | --- | --- | --- | --- | --- | --- | --- |
|  |  |  |  | **V_cmax_** | **J_max_** | **V_cmax_** | **J_max_** |
| Rice | CT | WW | 25 | 165.0±9.6 | 186.1±13.2 | 183.0±17.2 | 179.3±12.4 |
| Rice | CT | WD | 25 | 108.4±4.6 | 120.3±17.5 | 146.8±7.9* | 115.1±18.4 |
| Rice | CT | WW | 38 | 251.2±6.2 | 202.7±14.1 | 275.7±15.2 | 188.9±11.1 |
| Rice | CT | WD | 38 | 152.8±4.8 | 214.1±6.6 | 224.2±3.2* | 180.3±16.6 |
| Rice | HT | WW | 25 | 100.7±10.0 | 121.8±7.7 | 89.0±7.5 | 108.5±4.5 |
| Rice | HT | WD | 25 | 60.3±2.9 | 70.0±3.5 | 61.2±8.6 | 74.1±6.6 |
| Rice | HT | WW | 38 | 260.0±11.1 | 204.6±18.2 | 303.1±15.3 | 201.7±18.8 |
| Rice | HT | WD | 38 | 141.6±29.5 | 117.9±17.3 | 159.2±26.0 | 112.2±16.8 |
| Wheat | CT | WW | 25 | 173.6±10.2 | 182.7±10.2 | 169.6±3.7 | 170.6±4.5 |
| Wheat | CT | WD | 25 | 169.3±7.4 | 201.6±7.6 | 178.1±0.9 | 169.0±1.4* |
| Wheat | CT | WW | 38 | 156.8±25.4 | 147.7±13.9 | 207.4±45.1 | 146.7±15.4 |
| Wheat | CT | WD | 38 | 99.3±21.1 | 123.0±19.5 | 150.2±22.5 | 124.9±9.4 |
| Wheat | HT | WW | 25 | 159.8±10.9 | 145.5±13.7 | 149.2±12.9 | 136.5±12.8 |
| Wheat | HT | WD | 25 | 98.2±12.5 | 94.3±2.8 | 93.0±12.3 | 96.1±4.5 |
| Wheat | HT | WW | 38 | 153.8±17.6 | 161.6±12.0 | 277.5±30.6* | 169.6±14.0 |
| Wheat | HT | WD | 38 | 142.9±9.0 | 156.3±14.9 | 220.5±23.4* | 146.7±9.6 |

**Table S2.** Comparison of the mesophyll conductance (g_m_, mol m^-2^ s^-1^) estimated from three different methods: Harley et al. (1992), Ethier and Livingston (2004), Yin et al. (2009) using the Rubisco kinetic parameters measured *in vitro* in the present study for rice and wheat at the two temperatures, 25ºC and 38ºC (own kinetics), with regard to the Rubisco kinetic parameters reported for tobacco by Bernacchi et al*.* (2001, 2002) at 25ºC and 38ºC, in rice and wheat plants grown at CT and HT, under WW and WD conditions, and measured at 25ºC and 38ºC. Values are means ± standard errors (n = 5). Asterisks denote statistically significant differences by Duncan analysis (*P* < 0.05) between the Rubisco kinetics used (i.e., own kinetics *vs*. Bernacchi kinetics) within the same method and treatment.

| **Species** | **Growth T**  **(ºC)** | **Irrigation Treatment** | **Measurement T**  **(ºC)** | **Own kinetics** | | | **Kinetics by Bernacchi**  ***et al.* (2001, 2002)** | | |
| --- | --- | --- | --- | --- | --- | --- | --- | --- | --- |
|  |  |  |  | **g_m_ Harley’s method** | **g_m_ Ethier’s method** | **g_m_ Yin’s method** | **g_m_ Harley’s method** | **g_m_ Ethier’s method** | **g_m_ Yin’s method** |
| Rice | CT | WW | 25 | 0.439±0.086 | 0.335±0.027 | 0.456±0.091 | 0.354±0.037 | 0.306±0.025 | 0.317±0.050 |
| Rice | CT | WD | 25 | 0.187±0.046 | 0.123±0.020 | 0.102±0.026 | 0.217±0.090 | 0.121±0.016 | 0.087±0.018 |
| Rice | CT | WW | 38 | 0.204±0.025 | 0.192±0.032 | 0.172±0.032 | 0.464±0.098* | 0.188±0.025 | 0.132±0.020 |
| Rice | CT | WD | 38 | 0.189±0.038 | 0.237±0.037 | 0.150±0.049 | 0.152±0.011 | 0.242±0.055 | 0.114±0.031 |
| Rice | HT | WW | 25 | 0.197±0.013 | 0.262±0.023 | 0.283±0.063 | 0.273±0.025* | 0.330±0.045 | 0.253±0.055 |
| Rice | HT | WD | 25 | 0.107±0.007 | 0.262±0.057 | 0.441±0.029 | 0.115±0.019 | 0.245±0.077 | 0.365±0.020* |
| Rice | HT | WW | 38 | 0.263±0.042 | 0.346±0.044 | 0.319±0.009 | 0.347±0.116 | 0.258±0.034* | 0.299±0.009* |
| Rice | HT | WD | 38 | 0.139±0.013 | 0.151±0.039 | 0.286±0.016 | 0.170±0.050 | 0.234±0.080 | 0.215±0.027 |
| Wheat | CT | WW | 25 | 0.198±0.004 | 0.184±0.011 | 0.199±0.005 | 0.203±0.010 | 0.196±0.017 | 0.170±0.005 |
| Wheat | CT | WD | 25 | 0.664±0.060 | 0.320±0.012 | 0.421±0.068 | 0.661±0.069 | 0.382±0.030 | 0.423±0.038 |
| Wheat | CT | WW | 38 | 0.121±0.012 | 0.097±0.013 | 0.089±0.017 | 0.218±0.044* | 0.219±0.076* | 0.109±0.023 |
| Wheat | CT | WD | 38 | 0.149±0.041 | 0.158±0.012 | 0.068±0.018 | 0.097±0.041 | 0.269±0.068* | 0.085±0.026 |
| Wheat | HT | WW | 25 | 0.143±0.009 | 0.173±0.022 | 0.157±0.010 | 0.137±0.007 | 0.181±0.019 | 0.136±0.009 |
| Wheat | HT | WD | 25 | 0.106±0.008 | 0.180±0.031 | 0.087±0.013 | 0.107±0.008 | 0.161±0.007 | 0.078±0.012 |
| Wheat | HT | WW | 38 | 0.158±0.020 | 0.308±0.023 | 0.116±0.024 | 0.258±0.061* | 0.231±0.032* | 0.141±0.033 |
| Wheat | HT | WD | 38 | 0.107±0.007 | 0.158±0.020 | 0.066±0.008 | 0.139±0.010* | 0.189±0.038 | 0.073±0.010 |
